# Smart homes: pioneering age-friendly environments in China for enhanced health and quality of life

**DOI:** 10.3389/fpubh.2024.1346963

**Published:** 2024-05-17

**Authors:** Ingy Shafei, Jyoti Khadka, Madhan Balasubramanian

**Affiliations:** ^1^College of Business, Government and Law, Flinders University, Adelaide, SA, Australia; ^2^College of Nursing and Health Sciences, Flinders University, Adelaide, SA, Australia; ^3^South Australian Health and Medical Research Institute, Adelaide, SA, Australia; ^4^Menzies Centre for Health Policy and Economics, School of Public Health, The University of Sydney, Sydney, NSW, Australia

**Keywords:** ageing, smart home, models of care, China, gerontechnologies, home care, older adults

## Abstract

Traditionally, China has been more reliant on a model of care that ensures older adults are cared for by family members. Whilst promoting the idea of older adults ageing in their own homes is essential, the provision of in-home care must shift from primarily relying on family caregivers to a model that places greater emphasis on gerontechnologies and enhanced healthcare service delivery. In this perspective article we argue for the adoption of a ‘smart home’ model in aged care in China. The smart home model argues for innovative technologies to older adult care, such as virtual support groups, video-conferencing, and electronic health records; assistive technologies that can safely maintain independence and assist with daily living such as sensors, wearables, telehealth, smart home technologies as well as interactive robotic technologies for mobility and cognitive support such as humanoid robots, rehabilitation robots, service/companion robots. The adoption and implementation of gerontechnologies have been slow, with only a handful of solutions demonstrating proven effectiveness in supporting home care. The utilisation of such digital technologies to support and enable older adults in China to age-in-place can bring a significant contribution to healthy ageing. Nonetheless, it’s crucial to focus on co-creating with end-users, incorporating their values and preferences, and enhancing training to boost the adoption of these gerontechnologies. Through a smart home model of care, China can age-in-place more effectively, leading to significant contributions to healthy ageing.

## Introduction

The global population is rapid ageing, prompting focussed efforts to ensure positive pathways toward healthy ageing. The World Health Organization (WHO) defines healthy ageing as “developing and maintaining the functional ability that enables well-being in older age” (1). Whilst improvements in public health and rapid medical advancements are contributing to increased life expectancy, a rapidly ageing population presents profound challenges and complex consequences to health systems (2, 3). Older adults often present with multiple chronic conditions, accompanied by an increased demand for healthcare services, which in turn contributes to increase health system costs. For examples in Australia, the projected health expenditure per person (older adults) will rise from $3,250 AUD in 2028–19 to $3,970 in 2031–32 and reach $8,700 in 2060–61 (4).

Across the Asia and Pacific regions, one in seven people were aged 60 years or older^1^, and projections indicate that by 2050, one in four people will fall within this age group (5, 6). According to a report by the Asian Development Bank, a few countries such as the People’s Republic of China, Sri Lanka, Thailand and Vietnam, the demographic transition to old age will happen even more rapidly, which will have significant implications for health systems, as well as social and economic consequences (7). This transition will have significant implication on how cities and communities are built and organised, health services are delivered, as well as how employment and social security support individuals (7). It is imperative to acknowledge and optimise the social and economic contributions of older adults as a means to enhance sustainability.

## A focus on China

**Figure 1:**
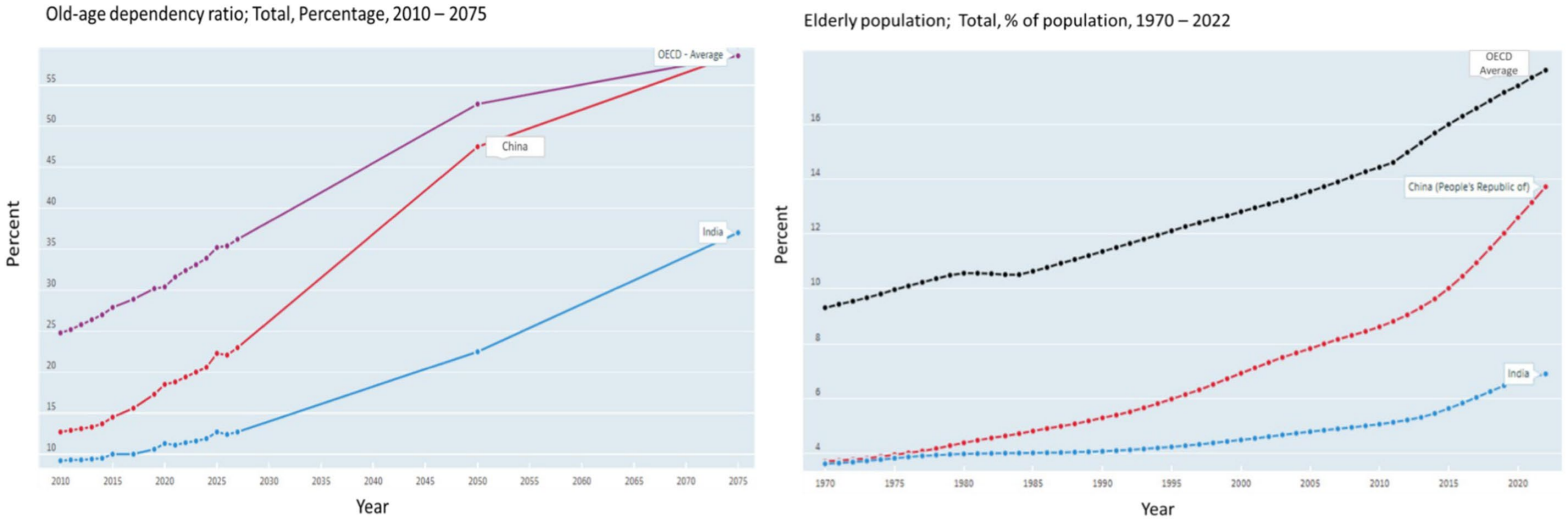
Old age dependency ratio; older adult’s population percent in China, India and OECD. Source: OCED (8).

According to the Organisation for Economic Cooperation and Development (OECD) data, older adults account for 13.7 percent of the overall population in 2022, accounting for nearly 280 million people (8, 9). This percentage is located between the OECD average (17.7 percent) and India (6.9 percent) in 2022 (see Figure 1). It is projected that the number of older adults in China will increase to 509 million representing approximately 40% of the total population in 2050 (5, 6). The old-age dependency ratio (number of individuals aged 65 and over per 100 people of working age between 20 and 64; see Figure 1) suggest that this ratio in China will reach that of OECD average around 2070 (approximately 58 older people for 100 people of working age). The increasing dependency ratio serves as a key indicator, emphasising the urgency for enhanced and efficient allocation of public resources and funding to address the requirements of the ageing population.

Traditionally, Chinese culture ensures older adults typically are cared for by family members (family care model), placing multitudes of burdens and facing myriads of challenges. From our viewpoint, we contend that the traditional model of family care for older adults in China is increasingly unsustainable. This model faces numerous challenges that affect both families and the healthcare system, primarily due to the swiftly evolving social and economic landscapes in China. Rapid urbanisation, demographic shifts toward an ageing population, and changes in family structures are placing immense pressure on the traditional model. These factors contribute to a growing burden on family caregivers and strain healthcare resources, underscoring the urgent need for innovative solutions and systemic changes to support the ageing population effectively. Whilst promoting the idea of older adults ageing in their own homes is essential, the provision of in-home care must shift from primarily relying on family caregivers to a model that places greater emphasis on technology (gerontechnologies) and enhanced healthcare service delivery. We argue a ‘smart home’ model will bring technology to assist in older adult care. Encouraging older adults to ‘age in place’ at home can help mitigate these challenges and alleviate the strain on health systems already operating at capacity in China [(10) p. 626 in (11)]. Utilising innovative digital technologies and smart homes can facilitate ageing-in-place in China. We explore a few international models and contemporary debates that advocate for the adoption of gerontechnologies aimed at facilitating healthy ageing, enhancing outcomes, and improving the quality of life for older adults in China.

## Traditional model and smart home solutions

In China, traditionally older adults have been living or cared for by family members (6). China’s older adults care has been described as ‘9073’: 90% by family, 7% by community care, 3% by nursing homes (12). With adult children typically expected to care for their older parents, public nursing homes typically welcome childless or poor older adults due to the perceived prejudice against utilising nursing home services in China (13).

Health expenditures in China have nearly tripled to 1,640 billion RMB from 2009 to 2018 (14). With urbanisation, economic growth, social transformation, smaller families, and limited nursing staff, the older adults are finding it increasingly difficult to rely on family members for support. The transition from a family-focused support system to technology-driven solutions, along with professional and community assistance, has extended the duration older adults can age in place within their homes (5, 6).

Long term care is a practise, and thus we need better informed solutions for home care. Smart solutions utilising technology can offer better integration with homes and health system practitioners and workforce. Long-term care (health and social) is defined by OECD as consisting of “a range of medical, personal care and assistance services that are provided with the primary goal of alleviating pain and reducing or managing deterioration in health status for people with a degree of long-term dependency, assisting them with their personal care (through help for activities of daily living (ADLs), such as eating, washing and dressing) and assisting them to live independently (through help for instrumental activities of daily living, IADL, such as cooking, shopping and managing finances)” (15) p.10.

Over the past decade, with older adults’ home care a policy priority, China has strongly promoted and committed to smart homes with the release of multiple comprehensive policies for older adults’ care. Firstly, in 2017, the State Council, the National Health and Family Planning Commission (NHFPC) and government agencies issued the “13th Five-year Plan for the Aging Development and Elderly Care Services” and the “13th Five-year Plan for Healthy Aging (2016–2020).” In addition, the Ministry of Industry and Information Technology and the Ministry of Civil Affairs issued the first national policy for smart homes “Action Plan for the Development of Smart Health and Elderly Care Services (2017–2020).” This was followed in 2021 by the renewed plan “Action Plan for the Development of Smart Health and Eldercare Industry (2021–2025)” (6, 13). In addition, the 14th Year plan for National informatization included actions to cultivate and expand smart older adults care services (16).

China’s older adults care promotes home-based care as the ‘cornerstone’ of care to be supported by community care, with long-term institutional care supplementing the former two when disabilities impact daily living (12). The 12th and 13th five-year plans come with a strong personal responsibility not just to have a healthy and happy life, but moreover from a civic duty perspective for health to avoid being a burden. The plans promote filial piety, older adults’ self-confidence, as well as advocating for older adults volunteering and entrepreneurial new business ownership, all contributing to healthy ageing. In addition, China invested in internet platforms, remote monitoring, virtual nursing systems, robotics and older adults care infrastructure across major cities, allocating over 740 million USD for pilot programmes for home-based older adults care (12).

## Smart home

Smart home solutions can play a significant role for in-home services. These include smart platforms making use of the internet of things (IOT), big data, mobile devices and cloud computing. Such technologies are revolutionising the industry and can have a significant contribution to facilitating ageing in place and meeting demands for home-based older adults care services (5, 6, 13). Innovations include information and communication technologies such as virtual support groups, video-conferencing and electronic health records; assistive technologies that can safely maintain independence and assist with daily living such as sensors, wearables, telehealth, smart home technologies as well as interactive robotic technologies for motility and cognitive support such as humanoid robots, rehabilitation robots, service/companion robots (17). The utilisation of such digital technologies to support and enable older adults in China to age-in-place can be remarkable and have a significant contribution for healthy ageing (13).

Monero et al.’s (18) conducted a systematic review on gerontechnologies designed to support ageing in place amongst home care residents and their caregivers. The review argues that the predominant use of these technologies was for monitoring older adults or their environments, improving communication with family caregivers, aiding in daily living activities, or delivering health information. Despite the wide range of applications, very few gerontechnologies were proven to be effective. The review strongly recommended the co-creation of gerontechnologies in partnership with end users to better accommodate their needs, values, and preferences, and highlighted the importance of incorporating training modules.

The development of smart home comes with its challenges and barriers for achieving large scale implementation. In 2016, the China Longitudinal Aging Social Survey of 11,000 older adults demonstrated that only 16% have a smartphone and less than 10% of actually use a smartphone (13), creating barriers to adoption for many technologies. A significant challenge for smart older adult care is in rural and remote China. Digital literacy is low to moderate at best, with digital devices deemed complicated and contributing to technological anxiety, even with family member support (19). In addition, the acceptance and willingness to learn of older adults is not high posing another critical limiting factor in technology adoption. Furthermore, price is a significant limitation, with smart home care technologies being quite costly in some instances further limiting technology adoption (19). The technology infrastructure, lack of regulation and development of services, as well as insufficient demand for services further compound the issue, also contributing to increasing costs (6). Finally, one of the key issues to cater the needs of older adults with cognitive decline which also acts as a significant barrier for use and adoption of innovative technology (19). Community older adult care is an innovative and much advocated older adult care model for China, with a consensus that this could contribute well to managing older adult care in China. In this model, older people do not need to move from their homes and social support in the form of community day care services is provided. These can be in the form of a visiting service in the home or a daycare centre for the older adult, with support provided at convenient times by family members when possible. Older adult’s people are still living within their familiar communities, thus contributing to healthy ageing in place and better outcomes for the older people (20).

Several factors have been advocated to influence adoption and implementation of smart older adult care, including affordable, price, user-friendliness, absence of operational difficulties, self-efficacy and confidence in utilisation of technology as well as the technology having clear benefits and advantages of use (13). In addition, financial inclusion and system stability, digital literacy and inclusiveness to close the digital divide is crucial. With significant central government support and expenditure as well as policies to guide and implement smart older adult care, the process of adoption can be accelerated for provision of smart home care services and technologies to address the rapidly changing ageing landscape in China success (19, 21).

Whilst various international studies and reviews (2, 13, 18, 19) have underscored the significance of gerontechnologies and the benefits of a smart home model, it’s crucial to develop such models with the specific needs and values of older adults and their families in China in mind. Paying close attention to the perspectives and requirements of the end users is key to creating a successful care model that can evolve gradually to support older adults and their family caregivers effectively.

## On reflection

Smart homes and gerontechnologies can lead to a sustainable solution for older adults in China and to ensuring healthy ageing and improved quality of life and utilising emerging technologies for smart homes will likely play a significant role in managing the care needs of rapidly ageing population. Strong infrastructure development and financial constraints are essential for successful policy implementation. Future research can further explore addressing the digital divide and demonstrate successful implementation of smart home solutions for older adults in China. It is also equally important to have evidence around the ethics and regulatory issues around the use of smart home technologies in aged care in China.

## Data availability statement

The original contributions presented in the study are included in the article/supplementary material, further inquiries can be directed to the corresponding author.

## Author contributions

IS: Conceptualization, Writing – original draft, Writing – review & editing. JK: Conceptualization, Writing – original draft, Writing – review & editing. MB: Conceptualization, Writing – original draft, Writing – review & editing.

## Footnotes

^1^It’s important to recognise that the classification of older individuals can vary based on age groups. Whilst in many developed countries, such as Australia, the threshold is set at 65 years and older, in most low and middle-income countries, individuals are typically considered older if they are 60 years and above.
